# Effects of Force Load, Muscle Fatigue, and Magnetic Stimulation on Surface Electromyography during Side Arm Lateral Raise Task: A Preliminary Study with Healthy Subjects

**DOI:** 10.1155/2017/8943850

**Published:** 2017-04-11

**Authors:** Liu Cao, Ying Wang, Dongmei Hao, Yao Rong, Lin Yang, Song Zhang, Dingchang Zheng

**Affiliations:** ^1^College of Life Science and Bioengineering, Beijing University of Technology, Beijing 100024, China; ^2^Medical Engineering Division, Xuanwu Hospital, Capital Medical University, Beijing 100053, China; ^3^Health and Wellbeing Academy, Faculty of Medical Science, Anglia Ruskin University, Chelmsford CM1 1SQ, UK

## Abstract

The aim of this study was to quantitatively investigate the effects of force load, muscle fatigue, and extremely low-frequency (ELF) magnetic stimulation on surface electromyography (SEMG) signal features during side arm lateral raise task. SEMG signals were recorded from 18 healthy subjects on the anterior deltoid using a BIOSEMI ActiveTwo system during side lateral raise task (with the right arm 90 degrees away from the body) with three different loads on the forearm (0 kg, 1 kg, and 3 kg; their order was randomized between subjects). The arm maintained the loads until the subject felt exhausted. The first 10 s recording for each load was regarded as nonfatigue status and the last 10 s before the subject was exhausted was regarded as fatigue status. The subject was then given a five-minute resting between different loads. Two days later, the same experiment was repeated on every subject, and this time the ELF magnetic stimulation was applied to the subject's deltoid muscle during the five-minute rest period. Three commonly used SEMG features, root mean square (RMS), median frequency (MDF), and sample entropy (SampEn), were analyzed and compared between different loads, nonfatigue/fatigue status, and ELF stimulation and no stimulation. Variance analysis results showed that the effect of force load on RMS was significant (*p* < 0.001) but not for MDF and SampEn (both *p* > 0.05). In comparison with nonfatigue status, for all the different force loads with and without ELF stimulation, RMS was significantly larger at fatigue (all *p* < 0.001) and MDF and SampEn were significantly smaller (all *p* < 0.001).

## 1. Introduction

Surface electromyography (SEMG) is a noninvasive technique to measure muscle electrical activity during muscle contraction, which can reflect the functional status of muscles. It has been widely used by clinicians as a diagnostics tool to identify neuromuscular diseases and disorders of motor control and to evaluate and monitor rehabilitation program [1].

SEMG is composed of action potentials from groups of muscle fibers organized into motor units (MUs) and therefore contains information about the characteristics and physiology of the active MUs [2]. The amount of force produced by a muscle depends on the MU activation patterns and the mechanical properties of the muscle fibers [3, 4]. Isometric contraction tasks such as handgrips have been applied to investigate the relationship between SEMG and force load of the upper limb [5, 6]. However, the handgrip task is not easy to perform for stroke patients with upper extremity movement disorder. Similar to the handgrip task, the side arm lateral raise task also generates isometric contractions, in which muscles generate tension without changing muscle length [7]. It is expected that performing the side arm lateral raise task could be easier in developing alternative rehabilitation programs to alleviate physical fatigue for stroke patients in comparison with handgrip task. This provides the clinical rationale of this preliminary study with healthy subjects.

Many physiological properties of the muscle, including the number of MUs, the peak discharge rates, and MU synchronization, are also affected by fatigue and peripheral stimulation [8, 9].

Muscle fatigue occurs after a prolonged or repeated muscle activity with a failure to maintain the required or expected force [10]. The degree of muscle fatigue can be measured by a relative maximal voluntary force loss during sustained contraction tasks [11, 12]. Muscle fatigue has been considered as one of the risk factors for musculoskeletal problems [13], which is one of the most difficult sequelae to adjust for many stroke patients who suffer from fatigue. Moreover, during rehabilitation process, fatigue may impair the patients' ability to regain muscle functions loss. Clinically, the perceived muscle fatigue has been used to evaluate the effectiveness of poststroke training program [14–16]. Although there was no clinically accepted indicator to assess fatigue, it has been reported that muscle fatigue leads to recognizable degradation of SEMG pattern [8]. It is therefore clinically useful to further investigate the relationship between muscle fatigue and SMEG feature change.

Low-intensity low-frequency magnetic stimulation has been shown to induce neuromodulation in humans without causing any pain [17, 18]. However, most of the previously published work applied the transcranial magnetic stimulation on the brain to alter human motor cortex excitability [19, 20]. It has been reported that extremely low-frequency (ELF, 3–30 Hz) pulsed electromagnetic field induced accelerated regeneration with injured peripheral nerves in rats [9, 21]. Although the peripheral magnetic stimulation has been studied recently, it has not been applied on human subjects [22, 23]. Therefore, the investigation on the effect of ELF magnetic stimulation on SEMG signal could provide preliminary evidence for a better understanding of the muscle activity.

Previous studies have investigated the separate relationships between SEMG signal and force and between SEMG features and neurophysiology of muscle fatigue [24–26]; however, there were no comprehensive studies to investigate the combinational effect of force load, muscle fatigue, and magnetic stimulation on SEMG, particularly during the side arm lateral raise task.

To analyze the changes of SEMG signal with muscle force and fatigue, various SEMG signal characteristics, including amplitude-based features, spectral features, time-frequency features, and nonlinear features of SMEG, have been analyzed during muscle contraction [27–29]. Root mean square (RMS) represents the signal power in the time domain and has been used to measure the level of activation of a muscle [8, 30]. Median frequency (MDF) is an indication of muscle fatigue in the frequency domain during isometric contraction [8]. It has been reported that the decrease of MDF with an increase in SEMG signal amplitude is a good indicator of fatigue [31]. As a measure of complexity due to the stochastic behavior of SEMG, sample entropy (SampEn) is related to the MUs recruitment and their firing rate [8, 24]. Moreover, RMS, MDF, and SampEn of SEMG signals have already provided meaningful evidence in association with physiological mechanisms during the muscle contractions [32, 33]. These features were therefore selected in this study, which in general reflect the amplitude, frequency, and nonlinear features of SEMG signals.

This study therefore aimed to quantitatively investigate the effects of different force loads on the RMS, MDF, and sample entropy derived from SEMG signals during the side arm lateral raise task and to compare the different effects between fatigue and nonfatigue status and between ELF magnetic stimulation and no ELF magnetic stimulation. The experiment will be conducted on healthy adults in this study to provide preliminary evidence for future development of alternative rehabilitation programs for alleviating physical fatigue.

## 2. Materials and Methods

### 2.1. Subjects

18 healthy male subjects (aged 25 ± 3 years) without any known history of neurological or psychiatric disorders were recruited. All subjects were right-handed, according to Oldfield's Edinburgh Inventory (Oldfield, 1971). Informed and written consent was obtained from each of the subjects after the aims, potential benefits, and risks were explained. The study was carried out according to the Declaration of Helsinki (1989) of the World Medical Association and approved by the Local Ethics Committee of Beijing University of Technology.

### 2.2. Experimental Procedure

**Figure 1:**
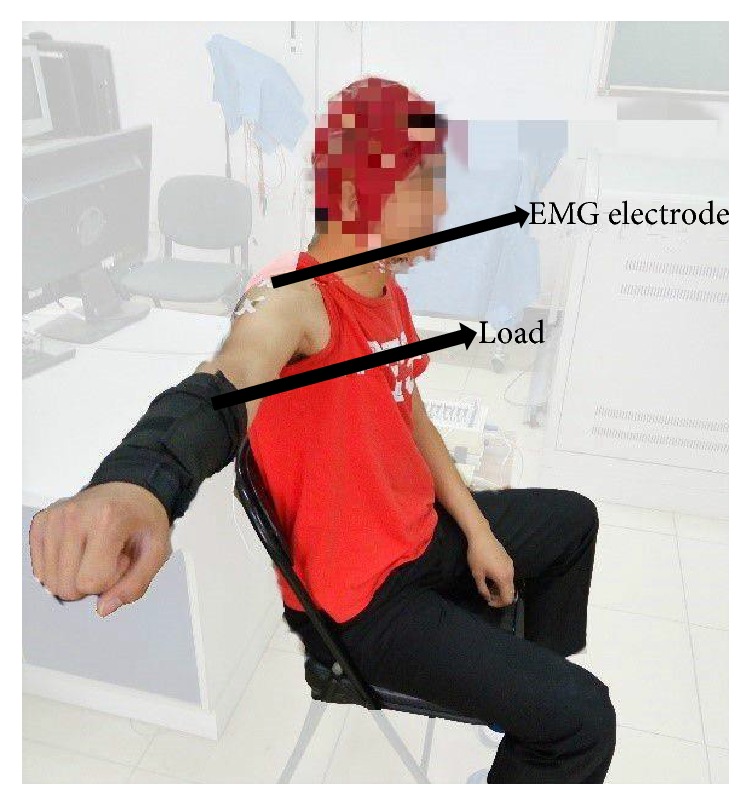
Illustration of lateral raise task with a subject sitting on a chair.

During the experiment, the subjects were asked to sit comfortably with the right arm side lateral raise (90 degrees away from the body) as shown in Figure 1. Different loads (0 kg, 1 kg, or 3 kg) were wrapped up on the forearm with a black bandage to generate isometric force at the upper limb muscle. The sequence of the loads was randomized among the subjects.

**Figure 2:**
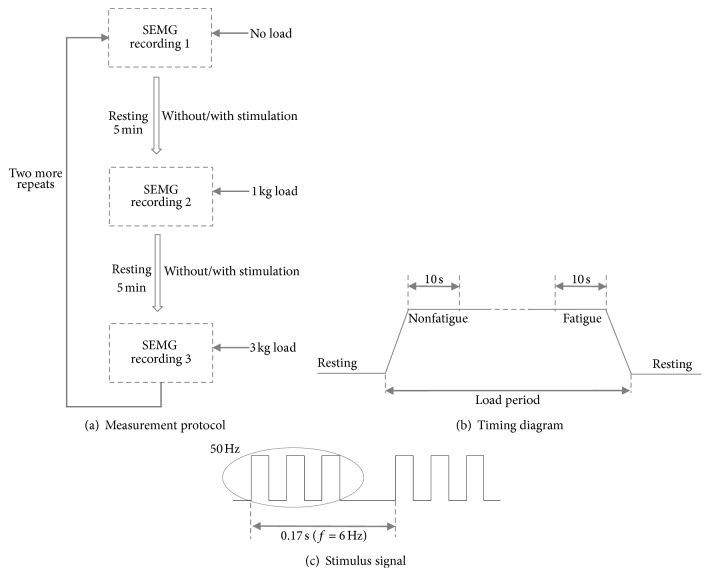
Measurement protocol, timing diagram of the recorded SEMG signal, and stimulus signal.

It has been suggested by clinicians that the anterior deltoid plays an important role in maintaining the lateral raise [7]. Therefore, SEMG signals were collected from the anterior deltoid of the right arm using flat-tape active electrodes attached to the skin. While the arm was laterally raised with a load, SEMG signals were recorded by a BioSemi ActiveTwo (BioSemi, Netherlands) system with a sampling frequency of 1024 Hz until the subject felt exhausted. The subject was then given a five-minute rest between different force loads. The same procedure was repeated three times with a total 9 SEMG recordings, as shown in Figure 2(a).

Two days later, the same experiment was conducted with additional 9 SEMG signals. This time, an ELF magnetic stimulation was applied to the subject's deltoid muscle during the five-minute resting period.

### 2.3. Magnetic Stimulation Device

The magnetic stimulation device was developed in our lab with a four-circular coil. The stimulus signal was generated and driven by an ARM microprocessor and power amplifier. The intensity and frequency of stimulation were adjustable between 10 and 40 mT and between 1 and 10 Hz, respectively. In this study, their corresponding values were 30 mT and 6 Hz. There were three 50 Hz pulses within each simulation cycle, and their duty cycle was 50%, as shown in Figure 2(c).

### 2.4. SEMG Signal Preprocessing

The recorded SEMG signals from a pair of electrodes were differentially processed. For the SEMG signal recorded at a certain load on the arm, the first 10 s recording was regarded as nonfatigue status and the last 10 s period before the subject was exhausted as fatigue status, as shown in Figure 2(b). The two segments of 10 s SMEG signals were then extracted for further analysis.

The interference (raw) EMG contains main frequency of 10~300 Hz [34, 35] and the low-frequency components of EMG are related to activation of a muscle [36]. Therefore, the surface EMG signals in our study were preprocessed using a 1~300 Hz band-pass filter and a 50 Hz notch filter to remove noise. Current source density transformations were then applied to reduce the effect of volume conduction on SEMG signals.

### 2.5. SEMG Feature Calculation

Three commonly used SEMG features (RMS, MDF, and SampEn) were calculated in this study.

#### 2.5.1. Root Mean Square (RMS)

RMS was calculated as

(1) $$\begin{array}{c} RMS=\sqrt{\frac{1}{n}\sum_{n}x_{n}^{2}}, \end{array}$$

where *x*_*n*_ is the value of SEMG signal and *n* is the number of samples. Here *n* = 2048 in this study.

#### 2.5.2. Median Frequency (MDF)

MDF is the frequency value that separates the power spectrum in two parts of equal energy [37]. It was calculated by

(2) fmed=imfsN,∑i=0i=imPi=∑i=imi=N−1Pi.

Power spectra density *P* was calculated by the method of averaged periodogram. The 10 s SEMG signal sequence (*x*(*n*), *n* = 0, 1, …, *N* − 1) was divided into *K* segments with *J* samples overlapping, and each of the segments had *L* samples. The recording was subdivided as *x*_*i*_(*n*) = *x*(*n* + *i*(*L* − *J*)), *i* = 0,1, …, *k* − 1, *n* = 0, 1, …, *L* − 1. In this study, *N* = 10240, *L* = 2048, *K* = 5, and *J* = 1024.

#### 2.5.3. Sample Entropy (SampEn)

Entropy is a nonlinear measurement of the complexity of SEMG signal. For a given embedding dimension *m*, tolerance *r*, and number of data points *N*, SampEn(*m*, *r*, *N*) is the negative logarithm of the probability that if two sets of simultaneous data points of length *m* have distance <*r* then the two sets of simultaneous data points of length *m* + 1 also have distance <*r*.

For the time-series SEMG of length *N* = {*x*_1_, *x*_2_, *x*_3_, …, *x*_*N*_} with a constant time interval *τ*, we defined a template vector of length *m* such that *X*_*m*_(*i*) = {*x*_*i*_, *x*_*i*+1_, *x*_*i*+2_, …, *x*_*i*+*m*−1_} and the distance function *d*[*X*_*m*_(*i*), *X*_*m*_(*j*)]  (*i* ≠ *j*). We counted the numbers of vector pairs in template vectors of length *m* and *m* + 1 having *d*[*X*_*m*_(*i*), *X*_*m*_(*j*)] < *r* and denoted them by *B* and *A*, respectively. The sample entropy was defined as

(3) $$\begin{array}{c} SampEn=-\log\left(\;\frac{A}{B}\;\right), \end{array}$$

where *A* is number of template vector pairs having *d*[*X*_*m*+1_(*i*), *X*_*m*+1_(*j*)] < *r* of length *m* + 1 and *B* is number of template vector pairs having *d*[*X*_*m*_(*i*), *X*_*m*_(*j*)] < *r* of length *m*.

The value of *m* was set to be 2 and the value of *r* was set to be 0.2 × standard deviation (SD) from 18 subjects at the same status. It could be seen from the definition that *A* has a value smaller or equal to *B*. Therefore, SampEn(*m*, *r*, *N*) has always either zero or positive value. A smaller value of SampEn indicates better self-similarity in SEMG.

### 2.6. Data and Statistical Analysis

The mean, standard deviation (SD), or standard error of the mean (SEM) of lateral raise task duration (the endurance time with a load until the subject felt exhausted) and the SEMG signal features (RMS, MDF, and SampEn) were calculated across all the subjects, separately for different force loads, for the fatigue/nonfatigue status, and for ELF magnetic stimulation/no ELF magnetic stimulation. Analysis of variance was performed using SPSS 22 (SPSS Inc.) to assess the measurement repeatability and the effect of force, fatigue, and magnetic stimulation on SEMG features, with their difference between forces, fatigue/nonfatigue, and stimulation/no stimulation compared. A *p* value below 0.05 was considered statistically significant.

## 3. Results

### 3.1. Lateral Raise Task Duration with Force

**Figure 3:**
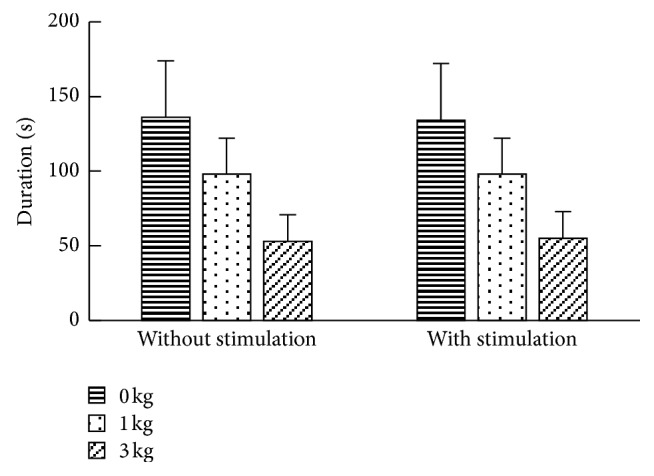
Lateral raise task duration with different force loads, separately for ELF stimulation and no ELF stimulation. The data was presented as mean ± SD.

The raise duration varied between subjects and with different force loads. As shown in Figure 3, the lateral task duration decreased significantly with the increase of force loads (*p* < 0.001). However, the duration was not significantly different for the same load with and without stimulation (*p* > 0.05).

### 3.2. Measurement Repeatability of RMS, MDF, and SampEn of SEMG

ANOVA analysis showed that there was no significant difference between the three repeated measurements for all the SMEG features derived in this study (all *p* > 0.05), demonstrating the reliability of the experimental setup. Therefore, the different features from the three repeated measurements were averaged for further analysis.

### 3.3. Effect of Force on RMS, MDF, and SampEn of SEMG

**Figure 4:**
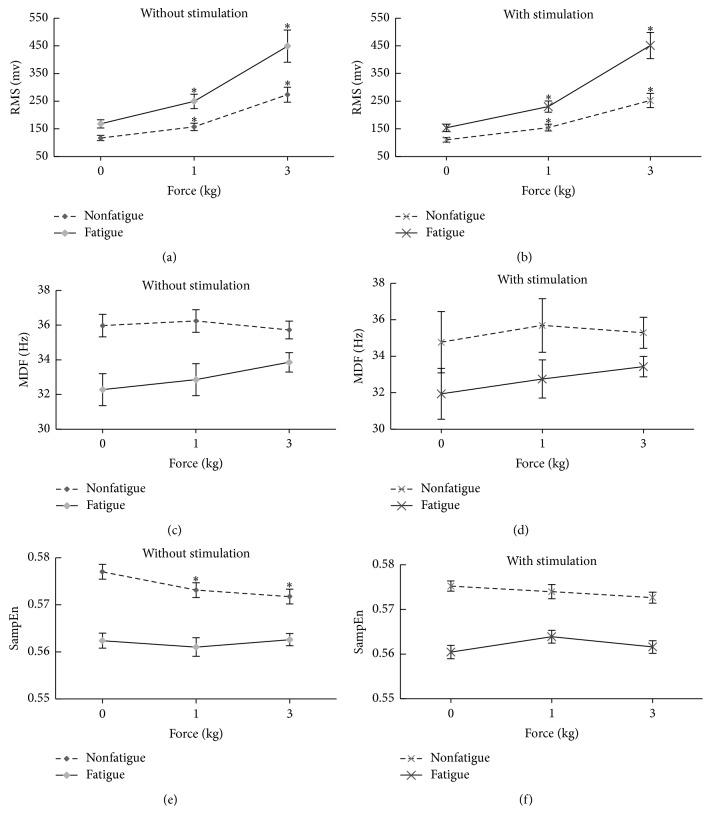
Mean ± standard error of the mean (SEM) of RMS, MDF, and SampEn with different force loads, separately for fatigue and nonfatigue and for ELF stimulation and no ELF stimulation. ^*∗*^Significantly different when compared with zero force.

ANOVA analysis showed that the effect of force loads on RMS was significant (*p* < 0.001). As shown in Figure 4, under both conditions (with and without magnetic stimulation), the RMS increased significantly with force at both nonfatigue status and fatigue status (both *p* < 0.001). SampEn decreased significantly with force only at nonfatigue status (*p* < 0.05). However, as a whole, the effect of force on MDF was not significant (both *p* > 0.05).

### 3.4. Comparison between Fatigue Status and Nonfatigue Status

**Figure 5:**
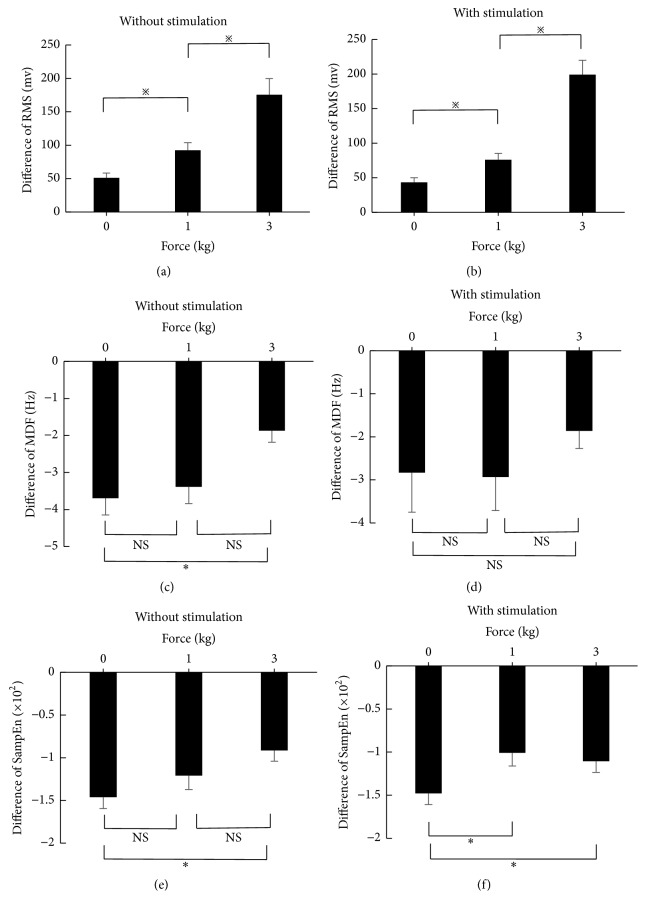
Differences (mean ± SEM of difference) of RMS, MDF, and SampEn of SEMG between fatigue and nonfatigue, separately for different force loads and for ELF magnetic stimulation and no ELF magnetic stimulation (^*※*^*p* < 0.001; ^*∗*^*p* < 0.05; NS: no significant difference).

The differences of RMS, MDF, and SampEn of SEMG signals between fatigue status and nonfatigue status are shown in Figure 5, separately for different force loads and for stimulation and no stimulation. Under both conditions (with and without ELF magnetic stimulation), the RMS at fatigue was significantly larger than nonfatigue (all *p* < 0.001), whereas MDF and SampEn at fatigue were significantly smaller than nonfatigue (all *p* < 0.001).

More importantly, the RMS difference between fatigue and nonfatigue gradually and significantly became larger with increasing load forces (both *p* < 0.001 for the comparison of RMS difference between 0 and 1 kg force and between 1 and 3 kg force loads), indicating that the force and fatigue had interactions on RMS. This has also been confirmed in the two-way ANOVA analysis where force and fatigue had significant interaction on RMS change (*p* < 0.001). However, there were no significant interactions for the MDF and SampEn of SEMG (both *p* > 0.05).

### 3.5. Comparison of Different Force and Fatigue Effects on SEMG Features with and without ELF Stimulation

The three SEMG features (RMS, MDF, and SampEn), their changes with force and their differences between fatigue and nonfatigue were not significantly different between ELF magnetic stimulation and no ELF magnetic stimulation (all *p* > 0.05).

## 4. Discussion and Conclusion

This study investigated the effect of force, fatigue, and ELF magnetic stimulation on SEMG signal features (including RMS, MDF, and SampEn) from the SMEG signals recorded with different force loads applied on the forearm during the lateral raised task. To the best of our knowledge, this is the first comprehensive study to quantify these effects.

As expected, the lateral raise task duration decreased with increased force loads on the arm. Although 60–70% of the subjects improved their endurance after ELF stimulation, the raise duration was not significantly different between magnetic stimulation and no magnetic stimulation. One of the possible reasons might be the fact that a larger sample size is needed in this experiment.

An objective and noninvasive assessment of muscle activity can be indicated by SMEG feature changes with different force loads. It is known that SEMG consists of the weighted sum of the electrical contributions of active MUs and therefore contains information about the characteristics and physiology of the active MUs including their activation and firing rates. During voluntary muscle contractions, the modulation of the firing rates of existing active MUs and the recruitment of new MUs are the two main mechanisms responsible for the maintenance of a specific level of force. Both the force exerted by a muscle and the amplitude of the SEMG depend on the number of recruited MUs and the discharge rate of each active MU. A higher muscle contraction level requires the recruitment of more MUs, resulting in higher RMS of the EMG signal [8, 30]. In this study, a statistically significant difference on RMS was demonstrated between different force levels. Therefore, our results agreed with physiological explanation with significantly increased RMS (*p* < 0.001) when the force load was increased. Additionally, it was found that there was no significant MDF difference between different force levels. Previous studies showed an increase in MDF of SEMG signals with an increasing level of muscle contraction [38]. However, those studies presented SEMG features at higher level of muscle contraction, and there are also discrepancies in opinions on MUs firing and recruitment at different levels of contractions. According to DeLuca and Erim's model, at a low level of muscle contraction, when a low number of MUs is recruited, the component of firing rates frequency in power spectrum density (PSD) is relatively high and more MUs are recruited, lowering the value of MDF [39]. Besides, force level did not affect SampEn of SEMG, indicating that the self-similarity of SEMG has not been changed with different forces. As far as we know, SampEn change with force level has not been studied before.

Muscle fatigue occurred when the subject was unable to maintain force during a sustained muscle contraction. Our work showed that RMS was larger at fatigue, and MDF and SampEn were smaller in comparison with nonfatigue status. This agreed with a published study [39], which explained that the newly recruited MUs, synchronization of MU firing, and decreased muscle fiber conduction velocity (MFCV) could be the possible mechanisms for increased SEMG signal amplitude at fatigue status [26, 40]. At the fatigue status, the drop in motoneuron excitability with sustained muscle activity results in decreased firing rates of active MUs [40] and slower MFCV, leading to MDF shift to lower frequency range. In terms of the results of SampEn, for a nonlinear measurement of the complexity of the signal of muscle fibers, the lower SMEG signal complexity may be related to an abnormal condition such as fatigue [29]. At fatigue status, with decreased firing rate of MUs, EMG signals have less stochastic behavior, leading to reduced SampEn. Previous study found that greater entropy corresponded to a broader power spectrum, and smaller entropy corresponded to a peaked power spectrum [29]. SEMG power spectrum becomes more peaked and concentrated in lower frequencies due to physiological mechanisms of muscle fatigue. Therefore, it appears that the entropy can be affected by a physiological mechanism similar to that which affects the median power frequency. This result is in accordance with the finding during isometric fatiguing contraction, where the entropy and median frequency decrease [41].

This study also showed that there was no significant difference in SEMG features and their changes with force between magnetic stimulation and no magnetic stimulation, which corresponded to the nonsignificant difference of raise duration with and without ELF stimulation. Some possible reasons could include the following: the intensity of ELF stimulation was too weak, the duration of stimulation was not long enough, or the ELF magnetic stimulation itself did not have delay effect on the SEMG signal recorded after the magnetic stimulation. Nevertheless, this study has provided preliminary evidence for future development of alternative rehabilitation programs for alleviating physical fatigue.

It is noticed that there was large interindividual variability due to different muscle strength between individuals. However, this preliminary study mainly focused on the within-subject comparison between loads, fatigue/nonfatigue, and stimulation/no stimulation. Besides, ANOVA analysis showed that there was no significant difference in all the SMEG features between three repeated measurements, separately for each load (all *p* > 0.05). Therefore, their averages from the three measurements were used for further analysis. In addition, for the two segments (two 10 s SMEG signals at nonfatigue and fatigue periods) used for signal processing, it was observed that the signals were quite stable without sharp baseline shift. A better way should be considered to control the experimental setup in further study.

One of the limitations of this study is that the task order (with and without simulation) should be randomized between subjects in the study design. However, it should be acceptable that the effect of the task order (with and without simulation) could be neglected in this particular study because there was a 2-days interval between the tasks.

Due to the variability of muscle characteristics between individuals, there is no simple way to define a precise muscle fatigue threshold. It is known that the amplitude of muscle contraction is often compared to maximum voluntary contraction (MVC), which can be rescaled to % of MVC. However, considering the potential clinical applications of raising arm, it may not be easy and completely safe to obtain the MVCs from patients with movement disorder. Therefore, to simplify the experimental procedure and reduce the study risks, as a preliminary study, the absolute forces were applied in this study. The absolute forces would impose different challenges between individuals, resulting in different duration of the lateral raise, as shown in Figure 3. The different effect of applying absolute force and % of MVC on both healthy subjects and patients could be comprehensively investigated in a future study. Additionally, other parameters with global perspective of the shifting in SEMG frequency may also demonstrate their association with muscle fatigue, leading to potential biological importance. For instance, SEMG power in gamma band (35~60 Hz) can also be investigated in a future study.

In addition, the effect of using different stimulus modes including the waveform, intensity, and frequency could be investigated, as well as the comparison with simultaneous SEMG recording during magnetic stimulation. Finally, as a pilot study, only male subjects were used. In the future, a comparison between male and female subjects is also worthy of further investigation.

In conclusion, our study comprehensively analyzed the effects of force, fatigue, and the ELF magnetic stimulation on SEMG features, which may facilitate better understanding of the underlying physiological mechanisms of muscle activities associated with force, fatigue, and SEMG response to ELF magnetic stimulation.

## References

[1] Wolf S. L., Butler A. J., Alberts J. L., Kim M. W. (2005). Contemporary linkages between EMG, kinetics and stroke rehabilitation. *Journal of Electromyography and Kinesiology*.

[2] Karlsson J. S., Roeleveld K., Grönlund C., Holtermann A., Östlund N. (2009). Signal processing of the surface electromyogram to gain insight into neuromuscular physiology. *Philosophical Transactions of the Royal Society A: Mathematical, Physical and Engineering Sciences*.

[3] Doheny E. P., Lowery M. M., FitzPatrick D. P., O'Malley M. J. (2008). Effect of elbow joint angle on force-EMG relationships in human elbow flexor and extensor muscles. *Journal of Electromyography and Kinesiology*.

[4] Anders C., Brose G., Hofmann G. O., Scholle H.-C. (2008). Evaluation of the EMG-force relationship of trunk muscles during whole body tilt. *Journal of Biomechanics*.

[5] Finneran A., O'Sullivan L. (2013). Effects of grip type and wrist posture on forearm EMG activity, endurance time and movement accuracy. *International Journal of Industrial Ergonomics*.

[6] Soo Y., Sugi M., Nishino M. Quantitative estimation of muscle fatigue using surface electromyography during static muscle contraction.

[7] Widmaier E. P., Raff H., Strang K. T. (2010). Muscle. *Vander's Human Physiology: The Mechanisms of Body Function*.

[8] Al-Mulla M. R., Sepulveda F., Colley M. (2011). A review of non-invasive techniques to detect and predict localised muscle fatigue. *Sensors*.

[9] Tasset I., Medina F. J., Jimena I. (2012). Neuroprotective effects of extremely low-frequency electromagnetic fields on a Huntington's disease rat model: effects on neurotrophic factors and neuronal density. *Neuroscience*.

[10] Gandevia S. C. (2001). Spinal and supraspinal factors in human muscle fatigue. *Physiological Reviews*.

[11] Rong Y., Hao D., Han X., Zhang Y., Zhang J., Zeng Y. (2013). Classification of surface EMGs using wavelet packet energy analysis and a genetic algorithm-based support vector machine. *Neurophysiology*.

[12] Rong Y., Moncel N., Zhang Y., Zhang D., Hao D. Fatigue muscle detection using time-frequency methods.

[13] Fimland M. S., Moen P. M. R., Hill T. (2011). Neuromuscular performance of paretic versus non-paretic plantar flexors after stroke. *European Journal of Applied Physiology*.

[14] van de Port I. G. L., Wevers L., Roelse H., van Kats L., Lindeman E., Kwakkel G. (2009). Cost-effectiveness of a structured progressive task-oriented circuit class training programme to enhance walking competency after stroke: the protocol of the FIT-Stroke trial. *BMC Neurology*.

[15] Prange G. B., Jannink M. J. A., Stienen A. H. A., Van Der Kooij H., Ijzerman M. J., Hermens H. J. (2010). An explorative, cross-sectional study into abnormal muscular coupling during reach in chronic stroke patients. *Journal of NeuroEngineering and Rehabilitation*.

[16] Houdijk H., ter Hoeve N., Nooijen C., Rijntjes D., Tolsma M., Lamoth C. (2010). Energy expenditure of stroke patients during postural control tasks. *Gait and Posture*.

[17] Robertson J. A., Théberge J., Weller J., Drost D. J., Prato F. S., Thomas A. W. (2010). Low-frequency pulsed electromagnetic field exposure can alter neuroprocessing in humans. *Journal of the Royal Society Interface*.

[18] Amirifalah Z., Firoozabadi S. M. P., Shafiei S. A. (2013). Local exposure of brain central areas to a pulsed ELF magnetic field for a purposeful change in EEG. *Clinical EEG and Neuroscience*.

[19] Hakansson N. A., Hull M. L. (2009). Muscle stimulation waveform timing patterns for upper and lower leg muscle groups to increase muscular endurance in functional electrical stimulation pedaling using a forward dynamic model. *IEEE Transactions on Biomedical Engineering*.

[20] Milanović S., Filipović S. R., Blesić S., Ilić T. V., Dhanasekaran S., Ljubisavljević M. (2011). Paired-associative stimulation can modulate muscle fatigue induced motor cortex excitability changes. *Behavioural Brain Research*.

[21] Das S., Kumar S., Jain S., Avelev V. D., Mathur R. (2012). Exposure to ELF- magnetic field promotes restoration of sensori-motor functions in adult rats with hemisection of thoracic spinal cord. *Electromagnetic Biology and Medicine*.

[22] Liu C., Zhu J., Li J. (2013). Functional magnetic stimulation system and pulsed magnetic-field effect on peripheral nerve. *IEEE Transactions on Magnetics*.

[23] Darabant L., Plesa M., Micu D. D., Stet D., Ciupa R., Darabant A. (2009). Energy efficient coils for magnetic stimulation of peripheral nerves. *IEEE Transactions on Magnetics*.

[24] Troiano A., Naddeo F., Sosso E., Camarota G., Merletti R., Mesin L. (2008). Assessment of force and fatigue in isometric contractions of the upper trapezius muscle by surface EMG signal and perceived exertion scale. *Gait and Posture*.

[25] Berchicci M., Menotti F., Macaluso A., Di Russo F. (2013). The neurophysiology of central and peripheral fatigue during sub-maximal lower limb isometric contractions. *Frontiers in Human Neuroscience*.

[26] Ravier P., Buttelli O., Jennane R., Couratier P. (2005). An EMG fractal indicator having different sensitivities to changes in force and muscle fatigue during voluntary static muscle contractions. *Journal of Electromyography and Kinesiology*.

[27] Hussain M. S., Reaz M. B. I., Mohd-Yasin F., Ibrahimy M. I. (2009). Electromyography signal analysis using wavelet transform and higher order statistics to determine muscle contraction. *Expert Systems*.

[28] Roman-Liu D., Konarska M. (2009). Characteristics of power spectrum density function of EMG during muscle contraction below 30%MVC. *Journal of Electromyography and Kinesiology*.

[29] González-Izal M., Malanda A., Gorostiaga E., Izquierdo M. (2012). Electromyographic models to assess muscle fatigue. *Journal of Electromyography and Kinesiology*.

[30] Gazzoni M., Farina D., Merletti R. (2001). Motor unit recruitment during constant low force and long duration muscle contractions investigated with surface electromyography. *Acta Physiologica et Pharmacologica Bulgarica*.

[31] Hagberg M. (1981). Work load and fatigue in repetitive arm elevations. *Ergonomics*.

[32] Meigal A. I., Rissanen S., Tarvainen M. P. (2009). Novel parameters of surface EMG in patients with Parkinson's disease and healthy young and old controls. *Journal of Electromyography and Kinesiology*.

[33] Phinyomark A., Quaine F., Charbonnier S., Serviere C., Tarpin-Bernard F., Laurillau Y. (2013). EMG feature evaluation for improving myoelectric pattern recognition robustness. *Expert Systems with Applications*.

[34] Moon H., Kim C., Kwon M. (2014). Force control is related to low-frequency oscillations in force and surface EMG. *PLoS ONE*.

[35] Neto O. P., Baweja H. S., Christou E. A. (2010). Increased voluntary drive is associated with changes in common oscillations from 13 to 60 Hz of interference but not rectified electromyography. *Muscle and Nerve*.

[36] Yoshitake Y., Shinohara M. (2013). Low-frequency component of rectified EMG is temporally correlated with force and instantaneous rate of force fluctuations during steady contractions. *Muscle and Nerve*.

[37] Kumar S., Mital A. (1996). *Electromyography in Ergonomics*.

[38] Bilodeau M., Arsenault A. B., Gravel D., Bourbonnais D. (1990). The influence of an increase in the level of force on the EMG power spectrum of elbow extensors. *European Journal of Applied Physiology and Occupational Physiology*.

[39] De Luca C. J., Erim Z. (1994). Common drive of motor units in regulation of muscle force. *Trends in Neurosciences*.

[40] Calder K. M., Stashuk D. W., McLean L. (2008). Physiological characteristics of motor units in the brachioradialis muscle across fatiguing low-level isometric contractions. *Journal of Electromyography and Kinesiology*.

[41] Xie H.-B., Guo J.-Y., Zheng Y.-P. (2010). Fuzzy approximate entropy analysis of chaotic and natural complex systems: detecting muscle fatigue using electromyography signals. *Annals of Biomedical Engineering*.

